# Effects of fibroblasts-derived exosomal FAP in regulating EMT in epithelial cells from chronic rhinosinusitis

**DOI:** 10.1016/j.bjorl.2025.101703

**Published:** 2025-09-06

**Authors:** Li-Fang Shen, Zi-Ming Fu, Hai-Hong Chen

**Affiliations:** Zhejiang University, College of Medicine, Department of Otolaryngology, Hangzhou City, Zhejiang Province, China

**Keywords:** Fibroblast activation protein, Exosome, Chronic rhinosinusitis, Epithelial mesenchymal transition, Epithelial cells

## Abstract

**Objectives:**

Exosomes play a crucial role in intercellular communication and may contribute to the development of various diseases. Nevertheless, their role in Nasal Polyps (NPs) remains poorly understood. Herein, Nasal Polyp Fibroblasts (NPF) were used to release exosomes, and epithelial cells were cocultured with NPF-derived exosomes to analyze Epithelial-Mesenchymal Transition (EMT) in Chronic Rhinosinusitis (CRS).

**Methods:**

We obtained exosomes from the culture media of NPFs treated with or without Fibroblast Activation Protein (FAP) inhibitor-4. Epithelial cells were coincubated with NPF-derived exosomes, and exosome internalization was analyzed by confocal microscopy. FAP and EMT markers in epithelial cells were detected by Western blotting and RT-PCR, while the migration ability of epithelial cells was detected by scratch experiments.

**Results:**

NPF-derived FAP-containing exosomes were rapidly internalized by nasal epithelial cells. These exosomes induced epithelial cells to express reduced E-cadherin and increased N-cadherin and vimentin levels, while FAPI-4 could reverse these changes. Furthermore, the migration ability of epithelial cells was enhanced by FAP-carrying exosomes. Our results reveal that FAP-containing exosomes promote EMT in epithelial cells.

**Conclusion:**

NPF-derived FAP-containing exosomes mediate the interaction between epithelial cells and fibroblasts and induce the EMT of epithelial cells, thereby potentially playing a crucial role in promoting remodeling in CRS. These findings suggest that FAP is a potential therapeutic target for the treatment of CRS patients.

**Level of evidence:**

Level 3 ‒ Non-randomized controlled cohort/follow-up study Recommendation B.

## Highlights

• NPF released more FAP-enriched exosomes upon stimulation of IL-36γ.
• The exosomes from NPF could be internalized by nasal epithelial cells.
• NPF-derived FAP-containing exosomes could induce the EMT of epithelial cells.

## Introduction

Chronic Rhinosinusitis (CRS) is closely linked to tissue remodeling.^1^ This remodeling process results in changes in epithelial thickness, vascularity, goblet cell hyperplasia, subepithelial fibrosis, and polyp formation.^2^ Dysregulation of Epithelial Mesenchymal Transition (EMT) can lead to organ fibrosis and has emerged as a new target for the clinical treatment of many chronic airway diseases related to tissue remodeling.^3^ EMT has been observed in CRS tissues, and is considered one of the key pathophysiological processes in CRS.^4^ Meanwhile, an aberrant EMT response may be a central factor in the early airway remodeling observed in patients with CRS.^5^

Fibroblasts are the primary producers of Extracellular Matrix (ECM), and have been reported to increase collagen and fibronectin deposition in Nasal Polyps (NPs).^6^ They also release inflammatory mediators, such as cytokines, chemokines and growth factors, which are involved in tissue remodeling. In addition, fibroblasts can inhibit the differentiation and proliferation of epithelial cells.^7^ Nishioka et al. found that fibroblast secretions in Chronic Obstructive Pulmonary Disease (COPD) can downregulate E-cadherin and upregulate vimentin expression in epithelial cells, thereby promoting EMT. While the interaction between fibroblasts and epithelial cells may play a crucial for EMT in COPD,^8^ there is currently no relevant research on how fibroblasts regulate the EMT process in epithelial cells. It has been shown that Transforming Growth Factor-Beta (TGF-β) induces EMT in both cancer cells and benign epithelial cells through the expression of Fibroblast Activation Protein (FAP),^9^^,^^10^ while inhibition of FAP can reduce TGF-β1-induced activation of pulmonary fibroblasts.^11^ Nonetheless, the role of fibroblast-epithelial cell interaction in CRS remains elusive. FAP, a key marker of fibroblast activation, is rarely expressed in healthy adult tissues, but is markedly upregulated during tissue remodeling and embryogenesis, including in conditions such as fibrosis, inflammation and tumors.^12^^,^^13^ This suggests that FAP may serve as a promising target and biomarker for related conditions. Nevertheless, no studies have yet investigated the role of FAP in CRS.

Exosomes can transfer signals from donor cells to recipient cells. Proteomic analysis of nasal mucosa-derived exosomes has revealed a variety of proteins and microRNAs associated with immunity, clotting, and tissue remodeling.^14^, ^15^, ^16^ However, the association between exosomes and the EMT process in diseases remains unclear, and no studies have investigated exosomal FAP in the secretions of patients with CRS. Wu et al. demonstrated that TGF-β1 mRNA was enriched in exosomes derived from glomerular endothelial cells treated with glucose, potentially mediating EMT and podocyte dysfunction.^17^ Li et al. discovered that the mouse alveolar macrophages highly expressed exosome containing miR-21-5p, which transferred miR-21-5p to the tracheal epithelium, and facilitated the EMT process.^18^ Exosomes derived from neutrophils may also contribute to airway remodeling in patients with severe and corticosteroid-insensitive asthma.^19^ These findings suggest that cellular communication via exosomes plays an essential role in the pathogenesis of these diseases.

Since non-Eosinophilic CRSwNP (non-Eos CRSwNP) is the predominant subtype in China, four patients with non-Eos CRSwNP were selected for this study. We aim to conduct research that reflects the characteristics of the Chinese population and provides relevant laboratory support for understanding regional differences in sinusitis. The criteria for determining non-Eos CRSwNP is an eosinophil count of fewer than 55 per high-power field and a proportion of eosinophils less than 27% of the total inflammatory cells. In the present study, we found that NPFs can produce highly concentrated FAP-containing exosomes when stimulated by IL-36γ. These exosomes were capable of transferring FAP to epithelial cells and ultimately promoting EMT. This is the first report demonstrating that NPF-derived exosomes play a key role in tissue remodeling associated with CRSwNP.

## Methods

The study protocol was approved by the Institutional Review Board, and written informed consent was obtained from each participant.

### Cell culture

NPs were collected from four patients with non-Eos CRSwNP during nasal surgery. Patients with malignancies, upper respiratory tract infections, or asthma were excluded from the study. Fibroblasts were isolated from the nasal polyp tissue. Specimens were cut into 1 × 1 mm squares and soaked in collagenase type IV (1 mg/mL; Sigma) at 4 °C overnight. The digested fragments were then transferred to a 0.25% Trypsin-EDTA solution and further digested at 37 °C for 15 min. The digested cells were then washed with PBS and suspended in DMEM containing 1% penicillin/streptomycin and 10% fetal bovine serum (FBS; Gibco, Life Technologies Corporation, Grand Island, NY, USA) in an incubator humidified with 5% CO_2_ at 37 °C. The purity of the sinonasal fibroblasts was confirmed by their characteristic spindle-shaped morphology. The human nasal epithelial cell line RPMI 2650 was obtained from the Cell Research Institute of the Chinese Academy of Sciences (Shanghai, China).

### Conditioned media collection

NPFs were divided into three groups: a control group (stimulated with PBS), an IL-36γ group (stimulated with IL-36γ at 150 ng/mL; CM77, Novoprotein, China), and an IL-36γ plus FAPI-4 group (treated with IL-36γ and FAPI-4; HY128643, MedChemExpress, China), with FAPI-4 used to inhibit FAP. NPFs were seeded in 6-well plates at a density of 2 × 10^4^/cm^2^ and treated with or without IL-36γ (150 ng/mL) for 24 h. Subsequently, the cells were cultured for an additional 24 hours in an exosome-free medium with or without FAPI-4. The conditioned medium was carefully collected and centrifuged at 1,000×g for 10 min, followed by a second centrifugation at 10,000×g for 15 min. Cleaned conditioned media with or without FAPI-4 were collected for further analysis.

### Exosomes isolation

The culture supernatant was collected and sequentially centrifuged at 300×g for 10 min, 2,000×g for 15 min, and 10,000×g for 30 min, 100,000×g for 70 min twice to pellet the exosomes. The purified exosomes were then resuspended in 200 μL of PBS.

### Nanoparticle tracking analysis (NTA)

A Nanosight LM10 system (Nanosight Ltd., Novato, CA, USA) was used to illuminate the nanoparticles and record their Brownian motion on video for 60 seconds. NTA was then performed on the video to measure the size and concentration of the nanoparticles.

### Transmission electron microscopy (TEM)

The exosome solution was dropped onto a copper grid and stained with 2% uranyl acetate. The grid was then washed, dried, and examined using a TEM (Hitachi, Tokyo, Japan).

### Western blot analysis

To analyze exosome proteins, exosomes from different groups were isolated using the same cell count. The samples were separated by SDS PAGE and transferred onto a PVDF membrane, which was then blocked with 5% BSA for 1.5 h. Primary antibodies were applied as follows: FAPα antibody (Abcam, ab207178; dilution 1:1000), CD63 (Abcam, ab134045; dilution 1:1000) and CD9 (Abcam, ab236630; dilution 1:1000) as exosomal marker proteins and GAPDH (GB12002, Servicebio, China; dilution 1:10000) as a loading control. Subsequently, the membranes were incubated with a secondary antibody (Servicebio, China) at room temperature for 2 hours. Bands were detected using an ECL kit (6300, CLINX, Shanghai, China).

### PKH67 labeling of exosomes

Firstly, the NPF’s exosomes (10 μg) were labeled using the PKH67 kit (Sigma-Aldrich) according to the manufacturer’s instructions.

### Uptake of the fluorescence-labeled NPF’s exosomes by epithelial cells

Epithelial cells were seeded onto 6-well plates and PKH67-labeled NPF-derived exosomes were added for co-incubation over 24 hours. The cells were then washed three times with PBS, and fixed overnight at 4℃ with 4% paraformaldehyde. After staining with DAPI nuclear stain (300 nM) for 3 min, the cells were observed under a confocal microscope.

### Co-culturing epithelial cells with NPFs-derived exosome and EMT markers detection

The in vitro experiment was divided into five groups. The epithelial cells without exosome treatment is the control group. In the second group, epithelial cells were incubated with PBS-treated NPFs-derived exosomes (PBS-NPFs-Exo group), it was the non-stimulated group. In the third and fourth groups, epithelial cells was incubated with IL-36γ-treated NPFs-derived exosomes (IL-36γ-NPFs-Exo 24 h group, IL-36γ-NPFs-Exo 48 h group), they were the stimulated groups. In the fifth group, epithelial cells were incubated with IL-36γ-treated NPFs-derived exosomes silenced for FAP (IL-36γ+FAPI-4-NPFs-Exo group), in this group, although fibroblasts were stimulated with IL-36γ, FAP expression was inhibited with FAPI-4.

Epithelial cells were seeded at a density of 2 × 10^4^ cells/cm^2^ in 6-well plates and treated with 2.5 mL of conditioned media without dilution for 24 h or 48 h.

Cell lysates (10 mg/each) were used to detect the expression of FAP, EMT markers by RT-PCR and Western blot. The Western blot procedure followed the method described above. Primary antibodies included the following: FAPα (ab207178, Abcam), E-Cadherin (20874-1-AP, Proteintech Group, Inc., USA), N-Cadherin (GB21390, Servicebio, China), Vimentin (GB11192, Servicebio, China), and GAPDH (GB12002, Servicebio, China). A secondary antibody from Servicebio (China) was also used.

Total RNA was obtained from samples using a TriZol reagent (Invitrogen). Superscript reverse transcriptase (Invitrogen) and oligo (dT) primers were used to synthesize complementary DNA. A StepOnePlus™ real-time PCR System (Applied Biosystems) was used to analyze FAP, EMT markers (E-cadherin, N-Cadherin, Vimentin), and GAPDH. The forward and reverse primers for the relevant genes are listed in Table 1.

**Table 1:** Primers used in quantitative real-time PCR reactions.

Gene	Forward primer (5’ to 3’)	Reverse primer (5’ to 3’)	Product length (bp)
FAP-α	TCTAAGGAAAGAAAGGTGCCAA	GATCAGTGCGTCCATCATGAAG	100
E-cadherin	AGTCACTGACACCAACGATAAT	ATCGTTGTTCACTGGATTTGTG	207
N-cadherin	CGATAAGGATCAACCCCATACA	TTCAAAGTCGATTGGTTTGACC	142
Vimentin	ACGCCATCAACACCGAGTTCAAG	GGATCTTATTCTGCTGCTCCAGGAAG	122
GAPDH	TTGGCTACAGCAACAGGGTG	GGGGAGATTCAGTGTGGTGG	264

FAP, Fibroblast Activation Protein.

### Cell migration assay

We assessed the influence of fibroblast-derived exosomes on the migration ability of epithelial cells using a cell migration assay. Epithelial cells were cultured with or without NPF-derived exosomes for 24 h, a 100 μL pipette tip were used to make multiple scratches. Images of the scratches were captured at 0- and 24 -hs, and the scratch area was analyzed using ImageJ software. Scratch Closure Percent (%) = (original scratch area-scratch area at measurement time)/original scratch area × 100%.

### Statistical analyses

All data are presented as mean ± Standard Deviation (SD). Statistical significance was determined using one-way ANOVA. All analyses were performed with SPSS 22.0 software (IBM, Armonk, NY, USA); p-values less than 0.05 were considered statistically significant.

## Results

### Characterization of exosomes derived from NPF

**Fig. 1:**
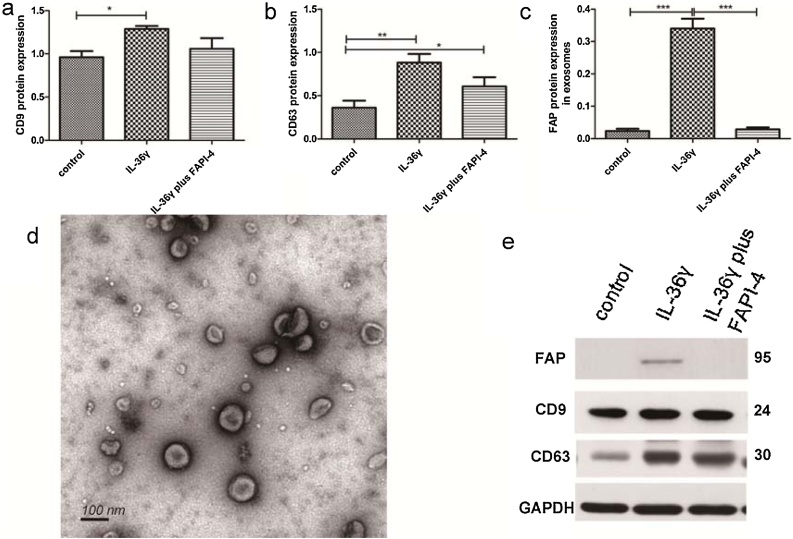
The NPF-derived exosomes was isolated and validated. Transmission electron microscopy showed the exosomes were round, membrane-bound vesicles. The exosome-enriched proteins CD9, CD63 and FAP were upregulated in IL-36γ group than in control group and IL-36γ plus FAPI-4 group, FAPI-4 could inhibit FAP expression. (The difference between groups was analyzed by one-way ANOVA. All data are presented as mean ± SD, *p < 0.05, **p < 0.01, ***p < 0.001).

The exosomes from each group of NPF were extracted separately. Both exosome marker proteins CD63 and CD9 were positively expressed (Fig. 1a, b and e). The exosome appeared as circular, membrane-bound vesicles with diameters ranging from 40 to 100 nm and a mean diameter approximately 80 nm, consistent with the characteristic of exosomes (Fig. 1d).

We further compared the number of exosomes secreted by NPFs with or without pretreatment with IL-36γ. The expression of CD9, CD63 was higher in IL-36γ group than in control group (Fig. 1a, 1b and 1e). Together, these results suggest that IL-36γ pretreatment can enhance the exosome secretion by NPFs.

### Increased expression of FAP in exosomes derived from NPFs

We found that FAP was upregulated in IL-36γ group compared to the control group and IL-36γ plus FAPI-4 group (Fig. 1c and 1e). This indicates that NPF-derived exosomes with IL-36γ pretreatment could express higher levels of FAP, and that FAPI-4 treatment can inhibit FAP expression in these exosomes.

### Fibroblast-derived exosomes are internalized by nasal epithelial cells

**Fig. 2:**
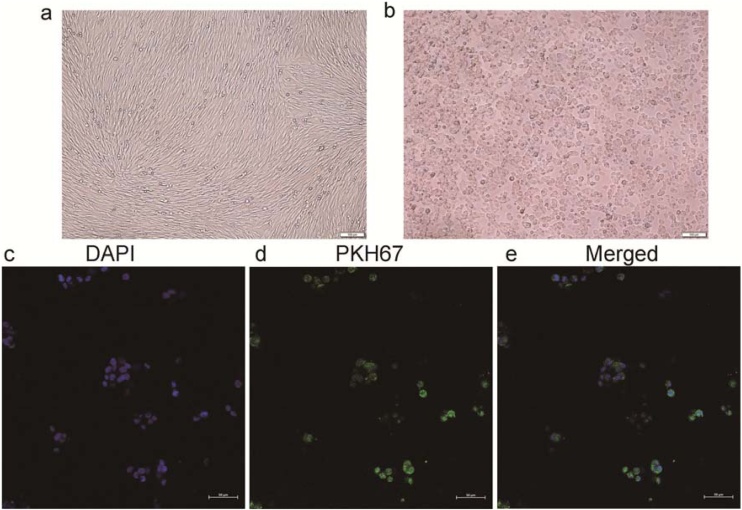
Confocal laser microscopy showed that PKH67-labeled exosomes were internalized by epithelial cells, and were localized to the perinuclear region of epithelial cells.

Exosomes and epithelial cells were stained with the green lipophilic fluorescent dye PKH67 and the blue nuclear dye DAPI, respectively. Exosome uptake by epithelial cells was observed as early as 3 hours post administration, with exosome accumulation in the recipient cells increasing over time. The PKH67-labeled exosomes were detected in the perinuclear region of the epithelium by confocal laser microscopy (Fig. 2).

### Uptake of the FAP-enriched exosomes from fibroblasts promotes EMT in epithelial cells

**Fig. 3:**
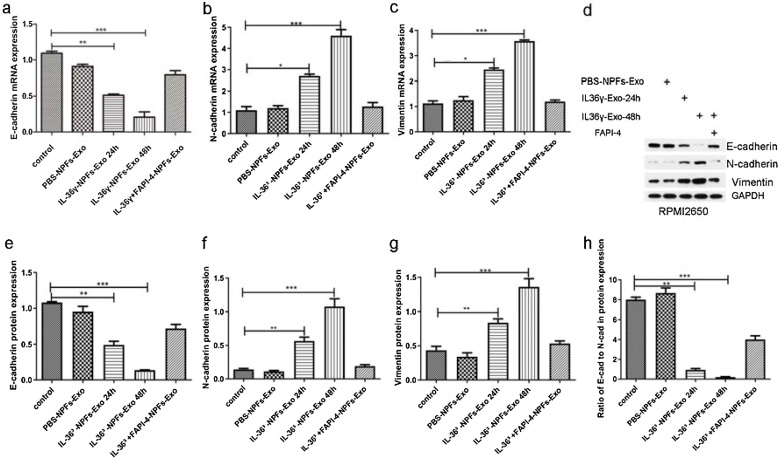
The expression levels of EMT-related markers were examined by Western blots and RT-PCR. (a, b and c) The results revealed that compared to the control gourp the mRNA expression of E-cadherin decreased and the mRNA expression of N-cadherin and vimentin increased in epithelial cells of IL-36γ-NPFs-Exo group, but not in epithelial cells of PBS-NPFs-Exo group, FAPI-4 inhibited this change. (d, e, f and g) Similar expression trends were obtained in protein expression of EMT-related markers. (h) The ratio of E-cadherin to N-cadherin was lower in epithelial cells co-cultured with NPF-derived exosomes pretreated with IL-36γ than in control group. (The difference between groups was analyzed by one-way ANOVA. All data are presented as mean ± SD, *p < 0.05, **p < 0.01, ***p < 0.001).

After co-culturing exosomes with epithelial cells, EMT-related markers in epithelial cells were assessed. The results showed that compared to the control group the protein expression of E-cadherin decreased, while the expression of vimentin and N-cadherin increased in epithelial cells co-cultured with NPF-derived exosomes from the IL-36γ-NPFs-Exo 24 h and IL-36γ-NPFs-Exo 48 h groups, but not in epithelial cells from the PBS-NPFs-Exo group. Importantly, FAPI-4 could inhibit these changes (Fig. 3). Similar expression trends were observed in the mRNA levels detected by RT-PCR analysis (Fig. 3).

The ratio of E-cadherin to N-cadherin, an indicator of the epithelial phenotype, was lower in epithelial cells co-cultured with NPF-derived exosomes pretreated with IL-36γ than in the control group, indicating increased EMT in the epithelium exposed to these exosomes (Fig. 3h).

### Effects of NPF-derived exosomes on the migration of epithelial cells

**Fig. 4:**
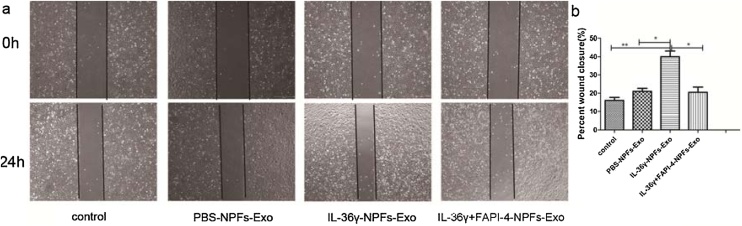
The effects of IL-36γ-NPFs-Exo, PBS-NPFs-Exo, IL-36γ+FAPI-4-NPFs-Exo on the migration ability of epithelial cells were detected by scratch test (×40) after 24 h. (The difference between groups was analyzed by one-way ANOVA. All data are presented as mean ± SD, *p < 0.05, **p < 0.01, ***p < 0.001).

The scratch assays revealed that the percentage of scratch closure in the IL-36γ-NPFs-Exo group was higher than in the control group, PBS-NPFs-Exo group and IL-36γ+FAPI-4-NPFs-Exo group. This indicates that the migration ability of epithelial cells was significantly enhanced after co-culture with NPF-derived exosomes pretreated with IL-36γ, (p < 0.05) (Fig. 4).

## Discussion

Although there is abundant evidence on the roles of epithelial cells and fibroblasts in airway remodeling, their interaction is not yet fully understood. Semlali et al. previously demonstrated that fibroblasts in asthma regulate the proliferation of epithelial cells.^20^ Meanwhile, Osei et al. suggested that abnormal communication between bronchial epithelium and fibroblasts is involved in the pathogenesis of COPD.^21^ Moreover, Nishioka et al. found that fibroblast secretions in COPD lung lesions can upregulate vimentin and E-cadherin expression in epithelial cells and promote EMT, indicating that fibroblast and epithelium interaction may contribute to EMT in COPD,^8^ however, there is no relevant research on how fibroblasts regulate EMT in epithelial cells.

Although, the association of FAP with EMT in cancer has been studied.^22^^,^^23^ No reports have revealed a link between FAP and EMT in inflammatory conditons.^24^ Our previous research detected FAP expression in NPs, revealing that FAP expression was negatively correlated with E-cad, and positively correlated with vimentin in NPs.^25^ FAP has been identified as a unique therapeutic target for the treatment of fibrosis.^26^ Some researchers consider FAP-expressing fibroblasts to be a distinct subpopulation associated with tissue fibrotic matrix remodeling.^27^ Therefore, in this study, we explored whether the activated fibroblasts can promote the EMT in CRS epithelial cells by secreting FAP and thereby participating in early tissue remodeling.

Exosomes serve as key mediators of intercellular communication in both pathological and physiological processes, such as immune regulation, extracellular matrix remodeling, and cell signaling.^28^ Due to these roles, exosomes have the potential to serve as diagnostic biomarkers and therapeutic targets for various diseases.^29^

Exosomes transport EMT-related proteins and miRNAs and may regulate EMT-related signaling pathways. Wu et al. revealed that the TGF-β1 mRNA is enriched in the exosomes from glomerular endothelial cells which may mediate podocyte dysfunction and EMT process in diabetic nephropathy.^17^ Studies have also confirmed that exosomes secreted by bronchial fibroblasts can promote EMT in airway epithelial cells.^30^ It has been demonstrated that exosomes derived from nasal lavage fluid can induce the migration of monocytes, neutrophils, and natural killer cells.^31^ Furthermore, exosomes from nasal lavage fluid and nasal epithelium in CRSwNP have been shown to inhibit the proliferation of human nasal epithelial cells.^32^ These findings suggest a high level of cell-to-cell communication within the NP microenvironment. Therefore, we reasonably predicted that exosomal FAP could exert a regulatory role on neighboring cells.

First, we stimulated fibroblasts with IL-36γ to establish a cellular inflammatory model and found that NPFs could secrete FAP-containing exosomes, fibroblasts stimulated with IL-36γ produced increased amounts of FAP-containing exosomes, while FAPI-4 inhibited FAP expression.

After co-incubation of epithelial cells with exosomes from the NPFs, numerous fluorescent puncta were observed in the cytosol, indicating effective exosome uptake. Next, FAP was delivered into the cells via the exosomes in a dose- and time-dependent manner, revealing that FAP-containing exosomes could promote EMT in epithelial cells in a concentration- and time-dependent manner. This was evidenced by changes in cellular morphology, decreased migration, down-regulation of mesenchymal markers (N-cadherin, vimentin), and up-regulation of epithelial marker (E-cadherin). These findings suggest that fibroblasts may induce EMT in CRSwNP epithelial cells by secreting FAP-containing exosomes.

Together, our findings suggest that cellular communication via exosomes plays an important role in disease pathogenesis. There is interaction between fibroblasts and epithelial cells in CRS, with exosomes acting as mediators in this process. In the future, exosomes may serve as effective carriers for drug delivery; for instance, exosomes carrying FAP inhibitors could potentially inhibit tissue remodeling in CRS and improve its prognosis.

Nevertheless, this study has several limitations. Firstly, we have only performed in vitro experiments and there is a lack of in-depth investigation into how FAP affects EMT in epithelial cells. Secondly, as we are still working to establish an air-liquid interface in vitro model, this study used RPMI 2650 non-differentiated cells submerged in culture media. This setup is less representative of the in vivo environment compared to an air-liquid interface culture. The in vitro model used may not fully reflect the influence of FAP on the respiratory mucosa, but rather the regenerative process or the reconstitution of the epithelial barrier. In the future, we plan to establish air-liquid interface in vitro experiments to improve our research. Thirdly, further studies are needed to explore the biological role of activated fibroblasts including relevant animal experiments. Nonetheless, we have proposed a new molecular mechanism by which fibroblast-epithelial fibroblasts and epithelial cells interact during EMT in CRS.

## Conclusion

The present study uncovered for the first time that NPF-derived. FAP can be carried and transferred by nasal exosomes, potentially mediating the interaction between epithelial cells and fibroblasts, and promoting tissue remodeling. Thus, exosomes carrying FAP inhibitors may inhibit the tissue remodeling in CRS and improve its prognosis. Collectively, our findings suggest that FAP is a potential therapeutic target for CRS patients in the future. However, the exact mechanisms by which NPF-derived exosomes facilitate EMT in the epithelium via FAP remain to be fully explored.

## ORCID ID

Zi-Ming Fu: 0009-0008-5597-1177

Hai-Hong: 0009-0004-9800-8752

## CRediT authorship contribution statement

The experiment was designed by LF Shen and HH Chen. LF Shen and ZM Fu carried out the test and analysis of the data. LF Shen collected and characterized the clinical specimens, and compiled the draft. All authors approved the final version of the manuscript.

## Ethics statement

The studies involving human participants were reviewed and approved by ethics committee of the First Affiliated Hospital, College of Medicine, Zhejiang University (IIT20220140B-R5). The patients provided their written informed consent to participate in this study.

## Funding

This research was supported by the Science and Technology Department of Zhejiang Province, China (nº LTGY24H130001).

## Data availability statement

The data that supports the findings are available on request from the corresponding author.

## Declaration of competing interest

The authors declare no conflicts of interest.
